# Draft Genome Sequence of Plesiomonas shigelloides MD22D9, Isolated from the Digestive Tract of Macrobdella decora

**DOI:** 10.1128/mra.00939-22

**Published:** 2022-12-14

**Authors:** Angeline Casale, Carlos Abudo Maldonado, Om Taropawala, Brandon O’Sullivan, Joerg Graf

**Affiliations:** a Department of Molecular and Cell Biology, University of Connecticut, Storrs, Connecticut, USA; University of Maryland School of Medicine

## Abstract

Here, we present a draft genome sequence of Plesiomonas shigelloides MD22D9, isolated from the digestive tract of the North American medicinal leech Macrobdella decora. The gut microbiome of the medicinal leech is hypothesized to be critical for maintaining host fitness. This genome can provide insights into this uncharacterized microbe-host relationship.

## ANNOUNCEMENT

Plesiomonas shigelloides is a Gram-negative, facultative anaerobe belonging to the family *Enterobacteriaceae* and is commonly found in brackish and freshwater ecosystems (1, 2). While P. shigelloides is a known opportunistic animal and human pathogen (2), its role in the medicinal leech is unknown (3–8). The gut microbiomes of sanguivorous leeches are hypothesized to be critical for maintaining host fitness and overcoming challenges of feeding exclusively on blood (9, 10). We present a draft genome sequence of P. shigelloides MD22D9, isolated from the North American medicinal leech Macrobdella decora, that could provide insight on an uncharacterized microbe-host relationship.

Plesiomonas shigelloides MD22D9 was isolated from a *M. decora* collected from a freshwater pond in Storrs, CT (41°49′3.6912″N, 72°15′33.588″W). The leech was anesthetized in 70% ethanol and subsequently sterilized with 10% bleach. The digestive tract was accessed with a sterile scalpel and intraluminal fluid was collected, plated on *Aeromonas* isolation agar (Sigma-Aldrich, St. Louis, MO), and incubated at 30°C. A single colony was streaked for isolation twice consecutively to obtain a pure culture before being stocked at −80°C in a glycerol solution.

For genome sequencing, the strain was streaked on LB agar and a single colony was used to inoculate LB broth following 18 h of growth (11). The MasterPure DNA and RNA purification kit (Lucigen, Middleton, WI) was used to extract genomic DNA. DNA quantification was performed using the Qubit 1× double-stranded DNA (dsDNA) high-sensitivity (HS) assay kit (Invitrogen, Waltham, MA). We used the Illumina DNA prep kit (Illumina, San Diego, CA) to create the genome library followed by 2 × 250-bp paired-end sequencing on the Illumina MiSeq platform using a MiSeq reagent kit V2 (500 cycles) (Illumina). Following sequencing, raw reads were first demultiplexed on BaseSpace and then downloaded.

The reads were trimmed (maximum ambiguities = 2, minimum accuracy = 95%, minimum read length = 15 bp) and assembled (minimum contig length = 200, word size = 21, bubble size = 241) *de novo* using CLC Genomics Workbench version 22.0 with default parameters (Qiagen, Hilden, Germany). The draft genome of P. shigelloides MD22D9 was 3,722,983 bp in size with a G+C content of 51.73%. It was assembled into 121 contigs with an *N*_50_ value of 311,795 bp and had an average coverage of 78.67× (Table 1).

**TABLE 1:** Genome summary and database accession information

Characteristic	Data for Plesiomonas shigelloides MD22D9
No. of raw reads	1,103,753
No. of reads in assembly	1,103,113
Genome size (bp)	3,722,983
Genome coverage (×)	78.67
No. of contigs	121
*N*_50_ value (bp)	311,795
G+C content (%)	51.73
No. of coding sequences	3,187
No. of protein-coding sequences	3,152
No. of tRNAs	95
No. of rRNAs	10
Closest neighbor, whole genome (%dDDH)	Plesiomonas shigelloides NCTC 10360 (88.3)
BioProject accession no.	PRJNA862914
BioSample accession no.	SAMN29999674
SRA accession no.	SRR20737870
WGS/GenBank no.	JANIFN000000000

The genome was annotated using the NCBI Prokaryotic Genome Annotation Pipeline version 6.2 (12). In total, 3,187 coding sequences were identified. Several genes implicated in virulence and symbiosis were found, including genes involved with the type VI secretion system (T6SS), motility, and heme/iron utilization (2, 10). Species-level identification was performed using digital DNA-DNA hybridization (dDDH) using TYGS version 342 (12). The closest genome to the subject strain was P. shigelloides NCTC 10360, with a dDDH of 88.3% when using the recommended d_4_ formula.

### Data availability.

The genome sequence of P. shigelloides MD22D9 has been deposited in DDBJ/ENA/GenBank under accession number JANIFN000000000. The version described in this paper is version, JANIFN010000000. The BioProject, BioSample, SRA, and whole-genome sequencing (WGS) accession numbers are provided in Table 1.
